# A machine learning approach predicts future risk to suicidal ideation from social media data

**DOI:** 10.1038/s41746-020-0287-6

**Published:** 2020-05-26

**Authors:** Arunima Roy, Katerina Nikolitch, Rachel McGinn, Safiya Jinah, William Klement, Zachary A. Kaminsky

**Affiliations:** 1 The Royal’s Institute of Mental Health Research, University of Ottawa, Ottawa, ON Canada; 2 Division of Thoracic Surgery, The Ottawa Research Hospital Research Institute and Ottawa University, Ottawa, ON Canada; 3 Faculty of Computer Science, Dalhousie University, Halifax, NS Canada; 4 Department of Cellular and Molecular Medicine, University of Ottawa, Ottawa, ON Canada; 5 Department of Psychiatry and Behavioral Sciences, Johns Hopkins University School of Medicine, Baltimore, MD USA; 6 Department of Mental Health, Johns Hopkins Bloomberg School of Public Health, Baltimore, MD USA

**Keywords:** Psychiatric disorders, Risk factors, Predictive markers

## Abstract

Machine learning analysis of social media data represents a promising way to capture longitudinal environmental influences contributing to individual risk for suicidal thoughts and behaviors. Our objective was to generate an algorithm termed “Suicide Artificial Intelligence Prediction Heuristic (SAIPH)” capable of predicting future risk to suicidal thought by analyzing publicly available Twitter data. We trained a series of neural networks on Twitter data queried against suicide associated psychological constructs including burden, stress, loneliness, hopelessness, insomnia, depression, and anxiety. Using 512,526 tweets from *N* = 283 suicidal ideation (SI) cases and 3,518,494 tweets from 2655 controls, we then trained a random forest model using neural network outputs to predict binary SI status. The model predicted *N* = 830 SI events derived from an independent set of 277 suicidal ideators relative to *N* = 3159 control events in all non-SI individuals with an AUC of 0.88 (95% CI 0.86–0.90). Using an alternative approach, our model generates temporal prediction of risk such that peak occurrences above an individual specific threshold denote a ~7 fold increased risk for SI within the following 10 days (OR = 6.7 ± 1.1, *P* = 9 × 10^−71^). We validated our model using regionally obtained Twitter data and observed significant associations of algorithm SI scores with county-wide suicide death rates across 16 days in August and in October, 2019, most significantly in younger individuals. Algorithmic approaches like SAIPH have the potential to identify individual future SI risk and could be easily adapted as clinical decision tools aiding suicide screening and risk monitoring using available technologies.

## Introduction

Suicide has been an intractable public health problem despite advances in the diagnosis and treatment of major mental disorders^1^. A growing area is the development of suicide screening technologies through accessing and analyzing social media data^2^. Previous studies have shown that youth are likely to disclose suicidal thoughts and suicidal risk factors online and on social media. For example, a study examining emergency room assessments of suicidality found that adolescents were likely to report suicidal ideations not only verbally, but also via electronic means, which included posts on social networking sites, blog posts, instant messages, text messages, and emails^3^. In addition, the authors reported an increase in the number of electronic communications of suicidality over the four year study-period, suggesting that this mode for expression of distress may become more common. Further, online expression of distress and suicidality may not be disclosed to physicians^4,5^. It is unclear to what extent such online expressions are comparable to suicidal risk as elicited by physicians, but some studies show a correlation of suicidal thoughts expressed online with psychometrically assessed suicidal risk^6,7^. Here we develop a machine learning approach based on Twitter data that predicts individual level future suicidal risk based on online social media data prior to any mention of suicidal thought. We then expand to a population level for validation and demonstrate that regional suicide rate data can be modeled by algorithmic scoring of randomly sampled Twitter data.

The past few decades have seen many advances in the diagnosis and treatment of mental health disorders, yet suicide continues to be a major health problem with annual suicide rates having been stable over the past 60 years at around 10–12 per 100,000^1^. Studies have demonstrated that many individuals prefer to be seen by their primary care practitioner for emotional problems, most likely due to reduced stigma and increased accessibility^8–12^. However, detection of suicide risk maybe low and around 60–70% of individuals at risk and seen by primary care practitioners prior to suicide attempts may go unidentified^13,14^. Further, a large number of at-risk patients may not receive appropriate care^15^. Therefore, cross sectional screening for suicidal ideation (SI) may be insufficient to capture those in need of intervention.

The National Action Alliance for Suicide Prevention identifies the development of biomarkers and predictive screening technologies as a priority area that would enable focusing limited resources onto individuals at risk^16^. A significant challenge in this area is the relative low base rate of suicidal behavior in the population, making prospective studies impractical. To address this, an area of growing interest is the development of suicide screening technologies through accessing and analyzing social media data. Of late, several research activities in this space have focused on accessing Twitter data^17^ as this platform has advantages for research; all data is public with users being advised to have no expectation of privacy and being informed that their data may be analyzed. Based on 2017 data in Canada, the largest portion of Twitter users were aged 18–35 years^18^. This age group represents 39–41% of the Canadian population^19^. Importantly, suicide is the 2nd leading cause of death in Canada in individuals aged 15–34^20^, suggesting that Twitter data may be ideal for the generation of novel tools to predict suicide risk.

In a systematic literature review, Pourmand et al. assert that youth and teenagers often disclose risk factors for suicide on Facebook and Twitter that they fail to disclose to physicians^21^. The authors conclude that tools enabling emergency departments to assess social media accounts could aid in clinical decision making^21^. Furthermore, in a cross sectional study of 1000 Twitter users in their 20’s, tweeting about suicidal ideation was significantly associated with assessments of suicidal ideation and behavior via self-reports^6^, suggesting that outcome metrics gathered through Twitter could represent a useful indicator of actual suicidal ideation and behavior. Twitter users who self-identify as having schizophrenia are more likely to tweet about suicide content^22^. Furthermore, Twitter represents a virtual location where users are known to make suicide pacts, searching for other users to join in a planned suicide attempt^23^. Followers of major media outlets are likely to retweet content related to health and disease, while content involving mental illness is associated with a higher probability of being retweeted^24^. Of all mental health related tweets shared by major media outlets, about 30% referred to suicide^8^.

A majority of publications that have harnessed information from social media have used Twitter data but unfortunately, what has been done is quite limited. Importantly, most studies assessed tweets which explicitly mention suicidal thoughts or attempts, but do not address tweets mentioning stress or other subtle signs of distress that are predictive of suicide risk. In a recent systematic review of publications in the application of technologies for suicide prevention, Franco-Martin et al. identified only 30 papers, of which 12.9% related to social networks and 3.23% were related to machine learning (ML)^25^. The authors concluded that, despite the promise of technological solutions to address and prevent suicide, much potential remains to harness such cutting-edge technological tools and predict suicide risk. Braithwaite et al. evaluated Twitter profiles of 135 study participants and validated a ML approach using the Linguistic Inquiry and Word Count (LIWC) approach to predict suicidal individuals with 92% accuracy^26^. In 2017, O’Dea et al. used LIWC analysis in Twitter data to demonstrate that ‘strongly concerning suicide related’ posts have distinctive linguistic profiles^17^. More recently in 2018, Du et al. have applied ML to generate a convolutional neural network capable of identifying suicidal tweets^27^. These methods identify tweets mentioning suicide and in doing so allow opportunities for prompt interventions in those at risk of suicide attempts. However, such approaches are limited in their ability to predict suicidal ideation prior to its development as it requires someone to have written a suicidal tweet in order for the model to identify it. Further, these approaches may not recognize suicidality in those who do not tweet about their ideations. Burnap et al. have developed a ML classifier to identify individuals with SI on Twitter with a classification accuracy of 68–73%^28^. After development of the tool, a 12 month application of the technique identified a binary classification of suicidal individuals with an accuracy of 85% as compared to human assessments by trained raters^28^. Importantly, it is unclear if the tool incorporates the expression of SI to generate predictive accuracy, which highlights an important gap in the field and a major opportunity for the application of AI in suicide prediction. Novel techniques should attempt to prognosticate not only who will be at risk for suicidal thoughts and behaviors but also when they will be at risk. To date, only one study has attempted to model temporal risk to suicidal thought through latent suicide topic analysis in 2016 Twitter data^29^; however, this study relies on an individual’s previous mentions of suicide topics to generate a personalized pattern and thus will be unlikely to identify individuals never having expressed suicidal thought in the past.

Our primary objective in this work was to generate a model capable of predicting individuals at risk as well as the odds of risk to suicidal thought within a given time frame. Our approach involved training ML techniques to measure existing patterns in tweets that are predictive of suicide risk but do not yet explicitly express suicidal thoughts. Second, ML techniques based on psychological theories of suicide are yet to be developed. Our approach centered on using ML constructs developed loosely around the interpersonal psychological theory for suicide (IPTS)^30^, the hopelessness model^31^, and associations of depression, anxiety, and insomnia with suicide risk. Research by Kleimen et al., has demonstrated an increased efficacy for accounting for suicide ideation variation through integration of multiple theories of suicide, demonstrating that hopelessness model scores were mediated to some extent by IPTS scores^32^, which supports the integration of multiple psychological constructs representing these theories. We hypothesized that algorithms integrating such approaches can offer nuanced and temporally sensitive tools to track and predict suicidal ideation in at risk individuals.

Here, we generate Twitter data based indicators of psychological constructs that are associated with SI and suicidal behavior using ML. We also apply ML to the generation of a cumulative indicator of suicide risk that predicts risk prior to the expression of suicidal ideation. Finally, we develop a temporal predictor of suicide risk periods and validate the technology on suicide decedents. We also provide an example of validation based on real-world data by using the technology to score and evaluate regional suicide rates. In the future, this tool, which we have termed “Suicide Artificial Intelligence Prediction Heuristic (SAIPH)”, could enable the suicide prevention community to screen for and monitor longitudinal changes in risk in or outside of care.

## Results

### Validation of neural networks

**Fig. 1:**
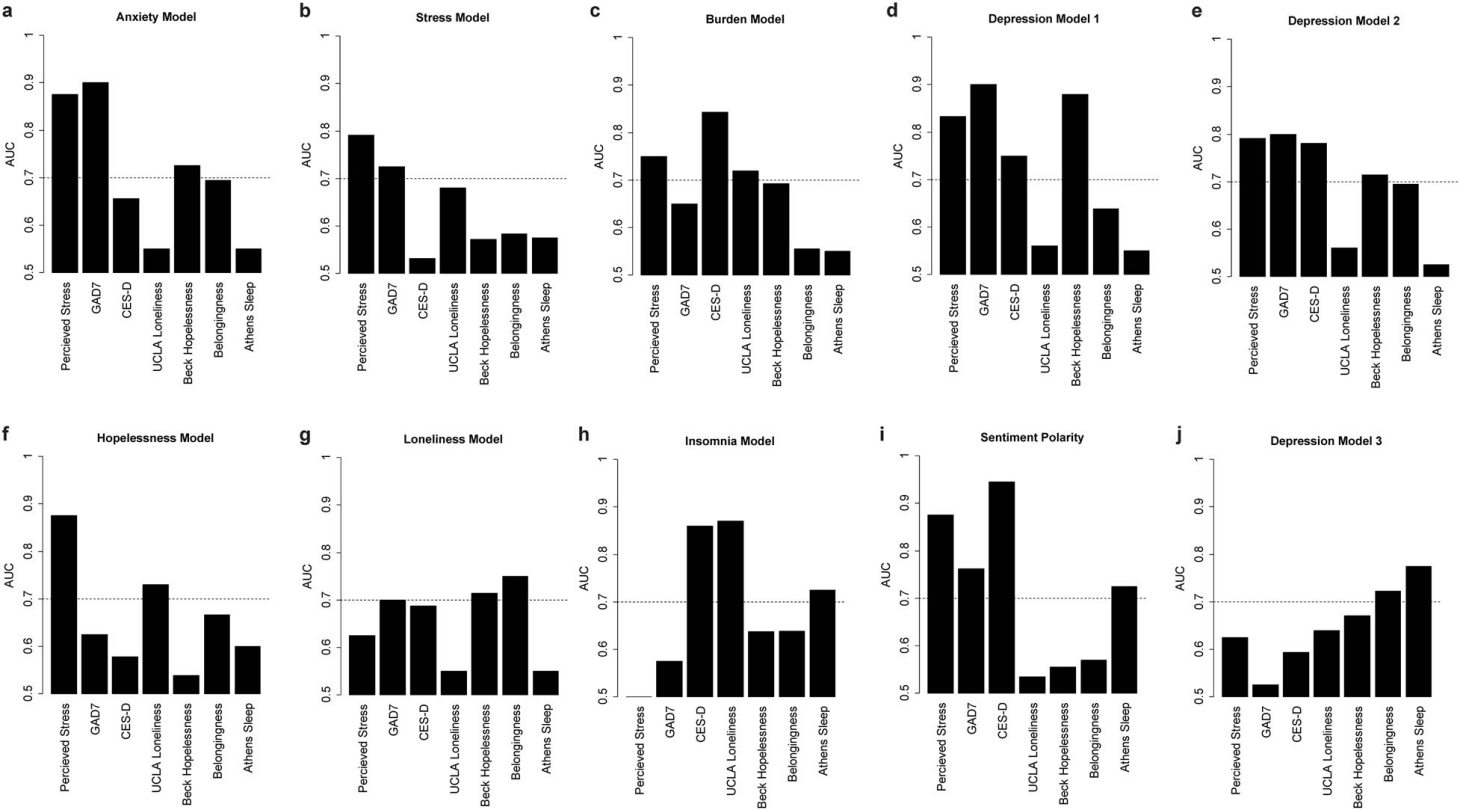
Neural network model performance to rate binary construct scales. Bar plots of the AUC of the ROC curve (*y*-axis) for neural network based classification of binary statement data adapted from various scales (*x*-axis) psychometrically validated to rate psychological constructs for the anxiety model (**a**), stress model (**b**), burden model (**c**), depression model 1 (**d**), depression model 2 (**e**), hopelessness model (**f**), loneliness model (**g**), insomnia model (**h**), sentiment analysis polarity metric (**i**), depression model 3 (**j**). A horizontal dashed line depicts an AUC of 70% accuracy. Binary adaptations of scales appear in Supplementary Table 3.

The neural networks constructed were designed to read text and infer psychological weights across a range of constructs including stress, loneliness, burdensomeness, hopelessness, depression, anxiety, and insomnia. Validation efforts against binary adaptations of psychometrically validated scales resulted in a range of accuracies for each neural network model, often with a given neural network exhibiting over 70% AUC to measure various psychological constructs (Fig. 1). We concurrently evaluated the polarity metric derived from sentiment analysis, determining that it most accurately measures depression, anxiety, perceived stress, and lack of sleep (Fig. 1e). Importantly, using our panel of nine neural networks, no psychological construct is unrepresented as each is accurately measured above 70% by at least one model. By comparison, support vector machines (SVMs) trained on the same bag of words as the neural networks and evaluated in the same manner appeared to be more specific in certain instances such as in evaluating sleep and loneliness (Supplementary Fig. 1); however, AUC levels generated by SVMs were significantly lower than that of neural networks (Student’s *T*, Mean NN: 0.68 ± 0.11, Mean SVM = 0.63 ± 0.12, *p* = 0.014) (Supplementary Fig. 2). For this reason, we chose to continue our model generation with the neural networks.

### AI model based prediction of suicidal ideation

Using 512,526 tweets from *N* = 283 SI cases and 3,518,494 tweets from 2655 controls (9.6% SI), we used the neural networks and sentiment polarity generated above to generate an array of ten metrics for each tweet. The resulting matrix was then used to train random forest models to predict a binary classification of SI case status using a bootstrap aggregating approach to address the imbalanced class ratio in model construction resulting in ten models. We attempted to validate it in *N* = 277 SI individuals relative to 2961 controls (8.4% SI). As such the proportion of SI in each set was similar to the observed rate of 9.2% observed in a cross-national study of SI rates in the general population^33^.

**Fig. 2:**
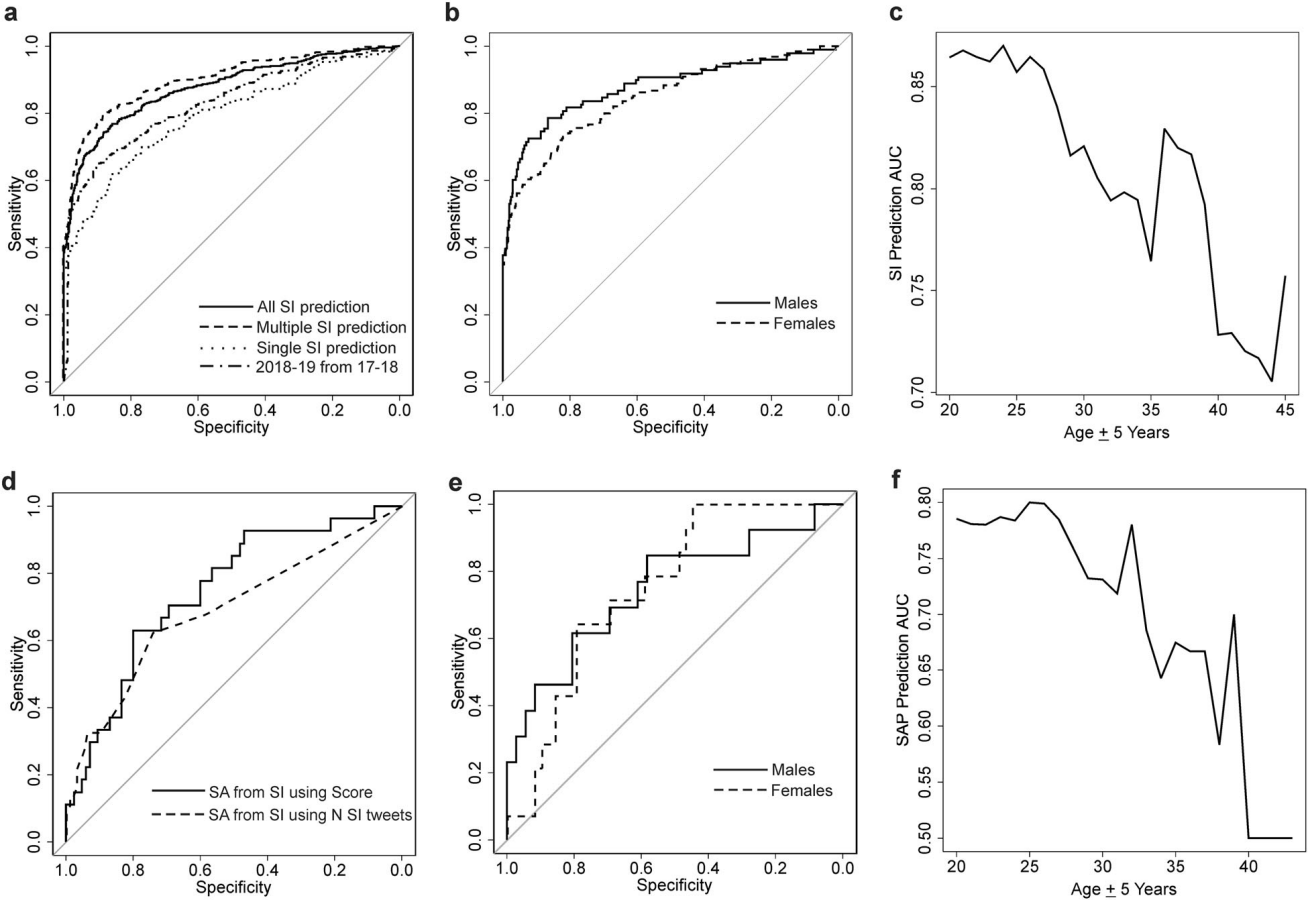
ROC curves for prediction of SI and SA. **a** Sensitivity (*y* axis) and specificity (*x* axis) is depicted for the prediction of individuals having expressed SI events as compared to controls in the in the original training and test sets across all individuals (straight line), individuals with multiple expressions of SI (dashed line) and those with only a single SI event (dotted line). Prediction of all SI individuals derived from August 2018 to May 2019 using models trained on data derived prior to August 2018 (variable dashed line). **b** Sensitivity (*y* axis) and specificity (*x* axis) is depicted for the prediction of individuals having expressed SI events as compared to controls in the in the original training and test sets across men (straight line) and women (dashed line). **c** The AUC of SI prediction (*y* axis) as a function of a sliding window of 10 year age groups centered on the *x* axis value (*x* axis). **d** Sensitivity (*y* axis) and specificity (*x* axis) is depicted for the prediction of individuals having expressed past suicide attempts or plans (SAP) from all non-SI individuals using the model score (straight line), from individuals with SI using the model score (dashed line), and from individuals with SI using the number of expressed SI tweets (dotted line). **e** Sensitivity (*y* axis) and specificity (*x* axis) is depicted for the prediction of SAP individuals from non-SI individuals in men (straight line) and women (dashed line). **f** The AUC of SAP status prediction (*y* axis) as a function of a sliding window of 10 year age groups centered on the *x* axis value (*x* axis).

Among the 277 individuals expressing SI, 132 expressed SI a single time while 145 expressed SI multiple times. We assessed the ability of the model to predict each of the *N* = 830 SI events derived from all 277 suicidal ideators relative to *N* = 3159 control events in all non-SI individuals using bootstrap aggregating across the ten models and averaging model output. The average model score from the period preceding the SI event by at least 1 day and at least 7 days following a previous SI even predicted SI events with an AUC of 0.88 (95% CI 0.86–0.90, Null AUC = 0.51 ± 0.0008, *p* = 0) (Fig. 2). This score was reduced to AUC = 0.80 (95% CI 0.75–0.84, *p* = 0, Null AUC = 0.52 + 0.019, *p* = 0) to predict those 132 SI events in single event suicidal ideators, and increased to AUC = 0.90 (95% CI 0.88–0.92, Null AUC = 0.51 + 0.0094, *p* = 0) to predict those 658 SI events in recurrent suicidal ideators (Fig. 2). By comparison, the mean sentiment analysis derived polarity score generated an AUC of 0.74 (95% CI 0.713–0.76, Null AUC = 0.51 + 0.0086, *p* = 0), while the bootstrap aggregated model trained with neural network scores but independent of sentiment analysis metrics generated an AUC of 0.85 (95% CI 0.83–0.87) to predict the *N* = 830 SI events derived from all 277 suicidal ideators relative to *N* = 3159 control events in all non-SI individuals. To assure that model performance was not driven by outliers in the test distribution, we generated AUC distributions from 10,000 permutations of randomly selected SI cases and controls of size *N* = 50 each (Supplementary Fig. 3). The resulting distributions were Gaussian and exhibited low variance, suggesting model performance was not influenced by outliers, that the neural network scores out perform sentiment analysis alone, and that the approach combining neural network and sentiment analysis derived scores attains the best results. Model sensitivity, specificity, and positive predictive value across different threshold scores are depicted in Supplementary Fig. 4. An inflection point maximizing sensitivity and specificity was identified at threshold of 0.683, generating a sensitivity of 80%, a specificity of 78.9%, and posterior probability of 42.4% increased risk of SI in individuals with scores above this threshold. As a proof of principle and to further validate our approach, we built a series of models using data derived only from prior to August 2018 in *N* = 265 SI individuals relative to *N* = 2422 controls and attempted to predict SI status in *N* = 278 SI individuals from *N* = 2911 controls collected from August 2018 to May 2019. These alternative models achieved and AUC of 0.83 (95% CI: 0.81–0.86, Null AUC = 0.51 ± 0.0098, *p* = 0) (Fig. 2a).

Model performance did not vary by sex, achieving an AUC of 0.88 (95% CI 0.83–0.92, Null AUC = 0.52 + 0.021, *p* = 0) to identify *N* = 125 SI tweet events from *N* = 1256 control events in males only and an AUC of 0.85 (95% CI 0.81–0.88, Null AUC = 0.51 + 0.015, *p* = 0) to predict *N* = 300 SI events from *N* = 937 control events in females only (Fig. 2b). Using a 10 year sliding window approach in estimated ages from 20 to 45 years old, we assessed SI prediction performance as a function of age. A significant negative correlation between age and model prediction AUC was observed (rho = −0.90, *p* = 1.42 × 10^−6^) (Fig. 2c). Notably, the age estimation algorithm only specifies ages under 18 and over 40 and as such, the tails of the interrogated age ranges should be interpreted with caution.

### Identification of individuals with past SA or suicide plan

We evaluated the algorithm’s ability to identify the most recent SI event from individuals with admitted past SA or suicide plan (SAP) from those with SI and no suicide attempt or plan (non-SAP). The algorithm predicted *N* = 37 SI events from SAP individuals from *N* = 97 SI events with an AUC of 0.75 (95% CI 0.64–0.85, Null AUC = 0.54 ± 0.044, *p* = 0) (Fig. 2d). Model sensitivity, specificity, and positive predictive value across different threshold scores are depicted in Supplementary Fig. 4. An inflection point maximizing sensitivity and specificity was identified at threshold of 0.731, generating a sensitivity of 80%, a specificity of 78.3%, and a posterior probability of 75.2% increased risk of an individual with SI being a SAP individual with a score above this threshold. We observed a significant association between the number of SI tweets across SI individuals with and without SAP status (Wilcoxon Rank Sum test: Mean non-SAP = 2.38 + 3.12, Mean SAP = 6.81 + 9.83, *p* = 2.8 × 10^−4^), which subsequently generated an AUC of 0.69 (95% CI 0.59–0.79, Null AUC = 0.53 + 0.04, *p* = 4 × 10^−4^) to identify SAP individuals from non-SAP suicidal ideators (Fig. 2b).

Model performance to predict the most recent SI event in SAP individuals relative to non-SAP individuals did not vary when evaluating males and females separately. The model achieved an AUC of 0.75 (95% CI 0.57–0.91, Null AUC = 0.57 + 0.065, *p* = 0.0082) to identify SI in *N* = 16 SAP males from *N* = 42 non-SAP males. In females, the model predicted SI in *N* = 21 SAP females from *N* = 55 non-SAP females with an AUC of 0.75 (95% CI 0.62–0.88, Null AUC = 0.56 + 0.058, *p* = 0.0018) (Fig. 2e). Using a 10 year sliding window approach in estimated ages from 20 to 45 years old, we observed a significant negative correlation between age and model prediction AUC (rho = −0.86, *p* = 8.25 × 10^−8^) (Fig. 2f).

### Temporal prediction of SI event

Suicidal ideation events and periods of suicide risk may be transient and so it becomes critical to identify not only who is at risk of suicidal ideation and more severe suicidal behaviors but also when they are likely to be at risk. To that end, we evaluated mean algorithm output data over a range of periods including 4, 7, 14, and 21 day spans per individual using a sliding window approach. We varied the start day from zero to 120 days (~four months) prior to the SI event. AUC values were negatively correlated with the time from the SI event (rho = −0.77, *p* < 2.2 × 10^−16^) with the highest AUC achieved at 1 day with a 21 day data span (AUC = 0.84, 95% CI 0.82–0.86); however, AUCs remained high (≥0.75) independent of starting position (Fig. 2a). To confirm this, we peformed a permutation analysis, randomly varying the start period per individual between 1 and 120 days and generated an average AUC of 0.805 ± 0.013 (*N* = 10,000 permutations). This suggests that while higher algorithm scores may indicate a period of more imminent risk, this implementation alone may not be adequate for determining the time of a likely SI event. In an attempt to improve model performance, we evaluated an alternate strategy whereby we counted the frequency of model scores above an individual specific threshold (denoted frequency score) as indicating risk, reasoning that an increased frequency of risk indicated scores may be indicative of increasing distress and might preceed an SI event. Frequency scores above an individual’s personal threshold (denoted peaks) exhibited increasing odds ratios (ORs) when they occurred temporallly closer to the time of the SI event (rho = −0.9, *p* < 2.2 × 10^−16^), with the most significant association occuring at 6 days from the SI event using a 21 day span (3 weeks) (OR = 6.1 ± 1.1, *p* = 6.9 × 10^−116^) (Fig. 2b). Notably, while less significant, the highest OR is found at 1 day prior to the SI event (OR = 6.7 ± 1.1, *p* = 9 × 10^−71^).

### Frequency score associates with timing of death by suicide

**Fig. 3:**
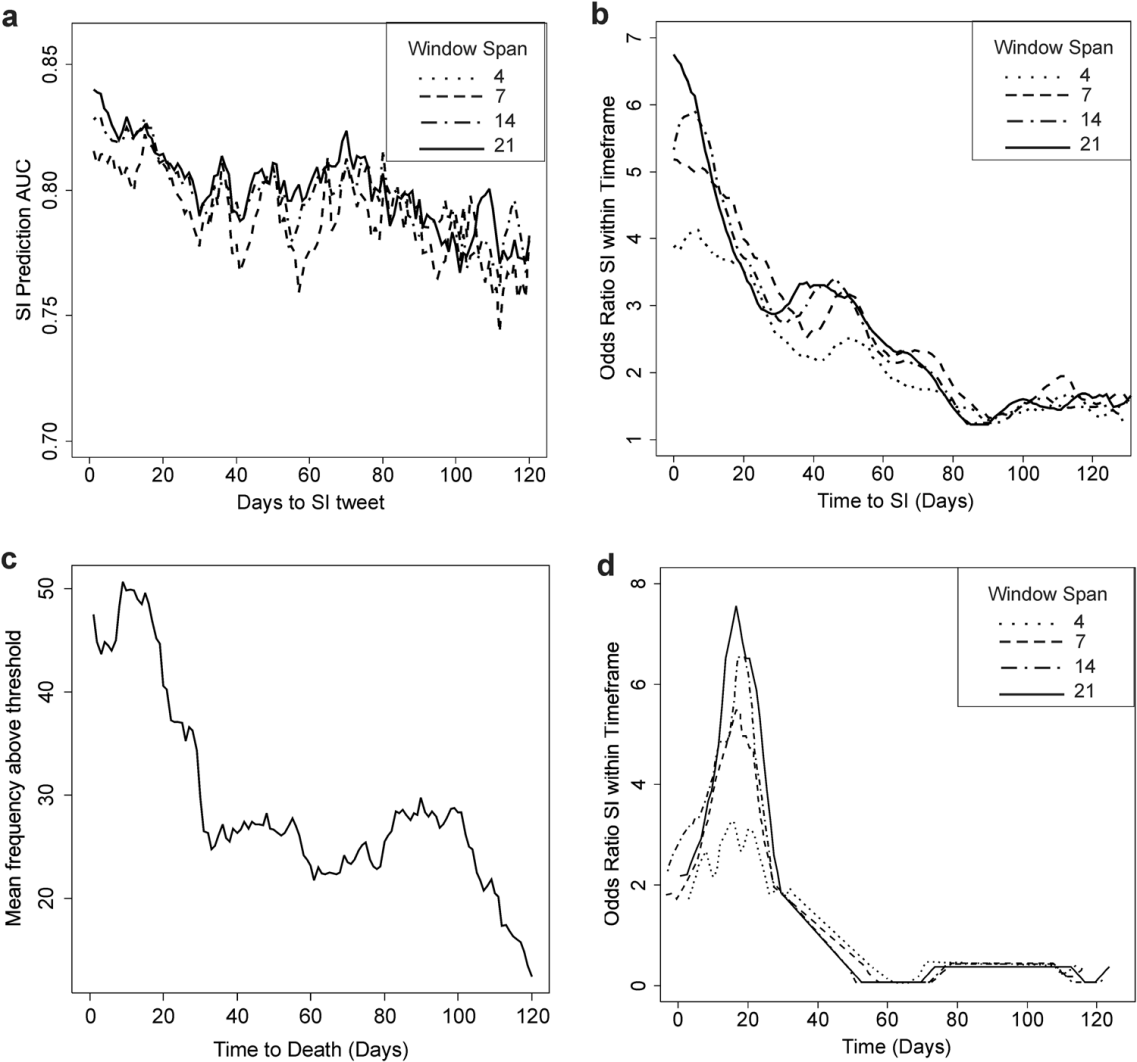
Temporal prediction of suicide risk. **a** A plot depicting the AUC of SI event prediction (*y* axis) as a function of the starting time from which tweet data was processed (*x* axis) for average model score data deriving from 4, 7, 14, and 21 days. **b** A plot depicting the OR that an SI event will occur (*y* axis) as a function of the starting time from which tweet data was processed (*x* axis) for frequency scores above each individual’s person specific threshold deriving from 4, 7, 14, and 21 days window analysis. **c** A plot depicting the mean frequency score per individual in *N* = 8 suicide decedents using a 21 day window span (y axis) as a function of time in days from death by suicide (*x* axis). **d** A plot depicting the OR of death by suicide (*y* axis) as a function of the starting time from which tweet data was processed (*x* axis) for frequency scores above each individual’s person specific threshold in *N* = 8 suicide decedents deriving from 4, 7, 14, and 21 days window analysis. Only significant logistic model data below a *p* value of 0.05 are depicted.

We analyzed the past four months of data from public Twitter profiles of celebrity suicides with our algorithm (Supplementary Fig. 5). After taking the average profile across all nine individuals using a sliding 21 day window, we identified a significant association of mean model scores with the time to suicide (rho = −0.70, *p* = 8.7 × 10^−19^) (Fig. 3c). We next assessed the period at which frequency score peaks associate with days from death using logistic regression and identified a region with a significant peak at approximately 20 days prior to death with the most significant association occurring with a window span of 21 days (OR = 7.6 + 1.2, *p* = 8.8 × 10^−20^) (Fig. 3d).

### Regionally generated SI scores model county-wide suicide rates

**Fig. 4:**
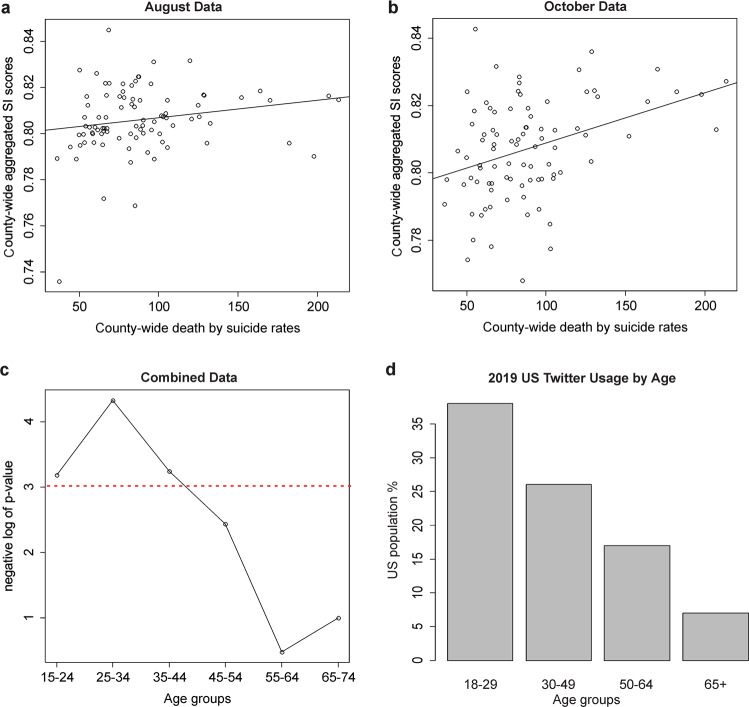
Association of SI score with county-wide suicide death rates. Scatterplots of the 2017 county-wide suicide death rate (*x* axis) as a function of the mean SI score of tweets collected within that county from a 16 day period in August (**a**) and October (**b**). A plot depicting the negative natural log of the *p* value of Kendall’s association between mean SI score per county (*y* axis) and the crude rate per age group (*x* axis) (**c**). The horizontal dashed red line depicts a *p* value of 0.05. A bar plot of the percentage of the US population using Twitter in 2019 based on data obtained from Statista (www.statista.com) (**d**).

To further validate our model, we assessed correlations between regionally associated SI scores randomly sampled across 92 US counties and Centers for Disease Control and Prevention’s (CDC) online database derived regional suicide death rates for the same regions. Fifty tweets were sampled and scored by our algorithm per county on an hourly basis for multiple days in August 2019. We observed a significant correlation of mean SI score per county with death rate (Kendall’s tau = 0.16, *p* = 0.021). A similarly significant result was obtained using regression analysis such that that aggregated SI scores were predictive of county-wide suicide associated death rate (IRR = 1.12, 95% CI 1.02–1.23, df = 91, *p* = 0.0201). We identified a significant association between population size-by-SI score interaction and death rates (Kendall’s tau = 0.38, *p* = 6.67 × 10^−8^). Similar results were obtained using hierarchical linear models to avoid the atomistic fallacy (Supplementary Note 1). We next used sub-samples of our data to understand the minimum number of days required to generate aggregated SI scores that would be predictive of death by suicide rates and determined a minimum of sixteen days was required (IRR = 1.91, 95% CI 1.30−2.82, df = 91, *p* = 0.0013) (Fig. 4a).

To validate our findings, we collected and scored additional Twitter data over twenty-nine days in September and October 2019 and observed significant associations between mean county SI scores and county-wide death rates (tau = 0.31, *p* = 1.16 × 10^−5^). Similarly, the hierarchical linear model analysis exhibited significant associations of SI scores with county-wide death rates over these twenty-nine days (IRR = 1.33, 95% CI 1.19–1.50, df = 91, *p* = 4.48 × 10^−6^) and the first sixteen days sub-sample (IRR = 1.24, 95% CI 1.07–1.44, df = 91, *p* = 0.0038) (Fig. 4b). Analyses restricted to the last sixteen days showed comparable results. Further sub-samplings showed that in this data from September/October 2019, SI scores over eight days were sufficient to predict county-wide death rates (multiple subsets of eight days; all *p* < 0.05). Evidence suggests that the discrepancy in the number of days necessary to significantly model regional suicide rates was driven by national response to mass shooting incidents occurring in early August 2019 (Supplementary Note 2), suggesting that model derived scores may be sensitive to acute but widely impactful events when analyzing short periods of time.

### Regional SI scores model age related county-wide suicide rates

We examined associations between our tweet derived SI scores and death by suicide rates for each age and sex group. Information on death by suicide rates was available for ages 5–74 years (in ten-year age groups) from the CDC online database. Significant associations were observed between SI scores of tweets collected over 49 days and county-wide death by suicide rates for age groups 15–24 (Kendall’s tau = 0.15, *p* = 0.041), 25–34 (Kendall’s tau = 0.18, *p* = 0.013), and 35–44 years (Kendall’s tau = 0.15, *p* = 0.038), but not for age groups 45–54 (Kendall’s tau = 0.12, *p* = 0.087), 55–64 (Kendall’s tau = 0.04, *p* = 0.620), or 65–74 years (Kendall’s tau = 0.08, *p* = 0.366) (Fig. 4c). Notably, the strength of the observed associations was comparable to the percentage of the US population using Twitter (Fig. 4d), and suggests that our sampled tweets derive from ages primarily below 45. No associations were found between SI scores and death by suicide rates in subgroups of males and females (data not shown).

## Discussion

Using a large data set of social media content collected over two years, we generated a series of neural networks to first generate proxy indicators of psychological state based on text that subsequently feed into random forest models to classify an individual as at risk for SI and those with a past history of SA or suicide plan from ideators. Using a training and test set of approximately equivalent sizes, a series of ten bootstrap aggregated random forest models integrating neural network model scores generated an AUC of 88% using tweet data from at least 1 day prior to the expression of SI by the case individuals. Importantly, the range of AUC values achieved using three weeks’ worth of data taken from any day within six months prior to the SI event averaged an AUC around 0.8, suggesting that this approach is efficacious for identifying individuals at risk for suicidal ideation that have not yet expressed such thoughts.

Our study also investigated the differences between individuals with multiple expressions of SI relative to those cases where only a single SI event was present in the data. As long periods of time, sometimes longer than a year, are available in the accessible data per individual, it is assumed that these single SI events have the potential to be the first expression of SI. The strength of the model to identify individuals with multiple SI events was higher than those with a single event. While we excluded data from 7 days after any previous SI event in multiple ideators to attempt to control for the influence of previous mentions of SI on subsequent predictions, it remains possible that residual signal from previous SI events was reflected in the text content after the excluded week and could account for the increase.

The model succeeded in distinguishing individuals with an admitted past history of suicide attempt or plan from suicidal ideators with an AUC of 0.75, suggesting that higher scores are indicative of more serious suicidal behavior. A caveat is that SAP status referred only to an admitted past history of suicide attempt or plan to attempt and thus, prospective studies will be required to assess the efficacy of the model to identify future risk to suicide attempt. One way to achieve this in future work may be to retrospectively collect and score Twitter timelines of individuals admitted to the hospital for suicide attempt to assess prospective predictive accuracy. In addition, we found a relationship between SAP status and the number of SI events expressed. While speculative, one possibility is that the frequency of expression of SI reflects a habituation to experiences associated with the concept of death and thus reflects the idea of acquired “capability” as expressed in the IPTS^30^ and idea to action framework^34^, and which is hypothesized as necessary in the progression from SI to SA. An alternative explanation is that those individuals that have survived a suicide attempt may be more likely to express instances of suicidal ideation relative to non-SAP individuals.

Using the M3Inference tool, we were able to infer the relative age and sex of those individuals comprising our data sets and determined that model performance was roughly equivalent for prediction of SI and SAP status in both males and females. As is reflected in the regional data analysis, the tool was more efficacious at predicting outcomes in individuals of younger ages. This result is expected and appears to mirror the Twitter usage statistics by age. In light of this, future studies seeking to generate social media AI tools for older individuals may benefit from a targeted representation of older users in the model training phase of the study.

A critical feature of our algorithm is not only the identification of who may be at risk for SI but when someone is likely to be at risk. We identified significant associations of higher peak frequency scores with an increased risk for more temporally proximal SI risk, with the most efficacious predictions deriving from three weeks worth of data. As the accuracy of identifying who may be at risk did not vary when analyzing data over a six month period, we envision a two-step approach, whereby high average SI model scores above a threshold may be used to indicate risk followed by an individualized analysis of the frequency of high scoring tweets relative to an individual’s specific tweeting pattern to identify when they are likely to be at risk. Based on the results of Fig. 3b, in individuals deemed to be at risk, identification of a peak over threshold would suggest the individual is seven times more likely to think about suicide in the next 10 days, while only 3 times or 1.5 times more likely within the following 40 and 80 days, respectively.

While identifying an increased frequency of risk tweets is intuitive for forecasting a period of impending risk, alternative machine learning techniques such as recurrent neural networks (RNNs) or long short-term memory (LSTM) models may represent promising areas of future research to model temporal risk to SI and suicidal behaviors. Despite their growing popularity in the machine learning and natural language processing fields, where they can be used to predict the next expected word from a text, for example, we opted to avoid these methods for the time being. Machine learning methods rely on two major assumptions including data representativeness of what is to be learned and that the various data distributions involved do not change over time^35^. In the first scenario, an increased risk state in someone experiencing SI for the first time may represent a rare event that challenges neural network-based forecasting due to class imbalance, where insufficient data points are present in the earlier data to represent the risk event that we want to identify. In natural language processing, this may be akin to asking the model to predict a word that has never occurred before in the text it has been trained from. Secondly, while RNNs would function effectively for discerning the next model state in the context of past model state, we felt they may not adequately capture external influences from a person’s environment that would cause fluctuations of data distributions over time. For example, someone may be having a wonderful day until they receive some bad news. At that point, tweeting patterns may change unexpectedly, just as stressors may influence precipitation of risk in the diathesis stress models of suicide. This in turn might violate the second assumption. Thus, while these models are likely efficacious for discerning patterns given a past history and might have been applied in each user as a means to gain insight into temporal dynamics, we reasoned that they may not be robust to the external influences occurring in a person’s life that alters their tweeting pattern. These types of changes may be abrupt, intense, complex, and most challenging to capture in a network that models the long term (as opposed to short term) interactions between features. Such are the limitations of deep learning methods, which have been shown to struggle to learn affect and behavior detection in education^36^. One promising approach warranting future study is the utilization of anomaly scores to model temporal rare events in imbalanced data. In 2019, Nguyen et al. employed a LSTM method on Twitter data to model disaster event detection, specifically looking for predicted future scores that exceeded error expectations and thus represent anomalies^37^. In a manner similar to our approach, the authors effectively used a downstream sliding window approach to assess model outputs for unexpected changes and demonstrated efficacy for the prediction of unexpected events. While future work should focus on evaluation of such techniques in suicidal behavior modeling at the individual specific level, performance is likely to vary as a function of the quantity of data available per person.

One potential weakness of a technology such as this one is that it is trained to identify a specific outcome which, in this case, is whether or not someone will tweet about SI. As mentioned above, SI expressions on social media have been linked to validated SI and suicidal behaviors^6^; however, despite this, we sought a way to further validate that our method has relevance to actual suicidal behaviors. To accomplish this, we first analyzed Twitter profiles of persons known to have died by suicide to assess individualized risk prediction and second took a population approach to assess the association of region model scores with regional suicide rates. In most suicide decedents analyzed, a frequency score risk peak was identified above the personalized threshold consistently within 20 days to death by suicide. Notably, the timing of risk peaks in individuals with SI occurs most proximal to the SI event; however, in people who died by suicide, statistical evidence suggests the most consistent peak occurs at 20 days. These observations are in line with studies that demonstrate a low proportion of people who died by suicide endorsed suicidal ideation prior to death^38,39^.

Using data from the CDC, we examined if our risk scores could be associated with actual death by suicide rates. These analyses demonstrated a link between county-wide aggregated risk based on tweets and death rates, suggesting that our model may be useful in predicting suicidal risk at a population level. We also found a moderating effect of the population size on these associations, suggesting that our algorithm may work better in a certain range of population sizes. However, more data with varied groups including a range of population sizes is needed to test this assumption before any conclusions can be drawn about the effectiveness of the algorithm at specific population sizes. We also found that cumulative risk scores over a minimum of 8–16 days were sufficient to predict a county-specific death rate. Preliminary analyses demonstrated that while an eight-day period was sufficient to predict death rates, this number increased during major events of national importance such as the Dayton and El Paso shootings. A caveat of these analyses is that we relied on the previous year’s death rates while using current Twitter-based suicidal risk to study the county-wide associations. While it can be argued that major year to year changes are not expected in county-wide death rates, nevertheless, this data is not reflective of the current status and therefore these results should be interpreted with caution.

Various efforts have been made in the past to link suicide related social media content to regional suicide rates. Using a weblog, posts from 2008 to 2010, Won et al. identified an association of suicide related content with monthly suicide death rate in South Korea^40^ after controlling for celebrity suicides, as these may be associated with changes in regional suicide rates^41^. In 2014, Jashinsky et al. analyzed 1,659,247 tweets for suicide key words and identified a correlation between the rate of risk tweet per US state and state age adjusted suicide rates^42^. In our work, the regions investigated were smaller in scale, suggesting that our technique may be scalable to smaller areas such as cities or neighborhoods within cities and should be investigated further. One potential application of these techniques could be in the evaluation of regional suicide prevention or postvention efforts in real time. The degree to which evaluating each will be efficacious remains to be studied; however, some evidence suggests that post-intervention evaluation may be more useful. By monitoring social media activity using autoregressive integrated moving average data analysis, McClellen et al. showed that suicide related tweet content increases following unanticipated events and lasts longer than anticipated events such as mental health awareness campaigns^43^. This data is consistent with our results, which, as mentioned previously, showed a significant change in mean county SI scores across the US in response to news of three mass shooting events in August 2019. Notably, the response to surprise events is complex, as Farhey et al. have noted in a study of 18 prominent suicides in Japan^41^, that the emotional content and degree of response on social media is contingent on the age, gender, and occupation of the deceased. This suggests that the manner in which our algorithm’s scores change during such incidents needs to be further studied. Importantly, the application of novel tools to evaluate regional changes in suicide risk will be important for optimization of suicide prevention and postvention practices.

Novel AI tools enabling the fast evaluation of suicide risk based on longitudinally collected data will facilitate suicide prevention efforts in multiple ways. Assessment of SI risk by computers could enable the efficient screening of individuals presenting in primary care settings and facilitate clinical decision making. Practically speaking, the time it takes from participant consent to data output is less than 30 s on average with our model on a standard Apache web server. Such tools could easily be scaled using local or web-based applications and accessed by clinicians on phones. An important consideration in such a context will be the tolerability of the intervention to false positives. As suicide and suicidal behaviors represent relatively low base rate events relative to the population, even very strong predictors will generate large numbers of false positives. Assuming the optimal sensitivity and specificity of the test identified above at 0.8 and 0.78, respectively, and a suicide ideation population rate of 10%, we will expect to identify 2.4 false positive suicidal ideators for every true one. The capacity to identify suicide attempters becomes more challenging, with approximately 53 false positive attempters identified for each true attempter. Therefore, were the medical community to rely on diagnostic tests alone, even those with promising performance metrics would soon add a likely intolerable burden to the health care system. As such, we envision the implementation of such tools as decision aids, giving gate keepers additional insight beyond a single point of care interaction by allowing an understanding of likely current state as well as an interpretation of that state in the context of the past. While the development of clinical decision aids at points of interaction with the health care system is important, such a view may be limited in scope. An alternative, complementary, and potentially more tolerable approach may be the devotion of health care resources directly to the online community. Novel predictive tools may enable identification of risk in individuals during a period of time prior to the progression of more severe suicidal behavior. Importantly, dissemination of suicide prevention information or redirection of individuals to professionally managed anonymous online suicide prevention resources will not add further burden and may even reduce burden to the health care system. Critically, the burden experienced by false positive individuals who receive suicide prevention related content may be tolerable such that this added benefit may outweigh the burden of targeting true positives. We speculate that our society has become accustomed to dismissing irrelevant ads. As one’s internet experience is generally performed one on one with a device, there is an assumption of privacy of the user experience. Future research will be necessary to assess if these methods will be tolerable to false positives and has the potential to reduce the burden of managing a phenomenon with a low base rate of occurrence in the population. Notably, a majority of suicide related content on Twitter involves the sharing of suicide prevention resources^44^. Furthermore, network analyses of Twitter users expressing suicidal ideation suggest a high degree of connectedness^45^, suggesting that influencing members identified online with suicidal ideation may have far reaching effects towards influencing other members with SI. Such methods should be studied and developed further and may represent a complementary arm to suicide prevention models such as the Nuremburg model, Zero Suicide, and other large scale community suicide prevention efforts like the OSPI in Europe^46^.

In addition to those mentioned above, a number of additional limitations of this study exist. The emphasis on depressive and stress based theories of suicide for inclusion in the design of the neural networks may represent a limited view that fails to capture additional risk trajectories leading to suicide. For example, substance use, which is a strong predictor of suicide risk in youth, was not included due to a relative poor performance of our neural networks to distinguish alcohol or substance use content from tweets. Other limitations include a lack of information regarding demographic and socioeconomic characteristics of the participants, specifically related to race, ethnicity, or nationality data. Understanding the efficacy of our model on varying populations will allow us to understand its equity and what populations may be underserved by future translational efforts. Additionally, linking both neural network and random forest output data to prospectively collected and psychometrically validated assessments will be important for validating the ability of these tools to track with changing mood state. These activities are planned for future studies. Another limitation is that the algorithm in its current form is limited to Twitter. While, in theory, the model can be applied to any text-based content such as Facebook posts and others, this has yet to be investigated. Another major caveat is the fact that the primary outcome is limited to the expression of SI on social media. While previous work suggests that admission of suicidal thoughts and behaviors on social media may be a useful indicator of suicidal behavior^6^, we cannot quantify the degree to which these expressions of SI equate to actions taken outside the context of social media. Furthermore, we have no ability to assess the proportion of control individuals that will have experienced SI without expressing it on social media. This fact may limit the potential translatability of the findings to prediction of expressions and experiences of SI independent of social forums. Additionally, control individuals were collected using the term “I” in a bulk fashion. While “event” days were selected at random in controls occurring in the same months as actual SI events in cases, a more robust control selection strategy may have been to collect controls using the query “I think” on the same day as collection of suicidal ideators. This strategy may have captured a similar “state of the world” comparison in controls and better controlled for any confounding effects of mood fluctuations due to world events like politics and economic changes, among others. Despite this, our demonstration that the algorithm significantly associates death by suicide in both individuals and using regional data adds confidence that our algorithm is detecting suicide risk. Importantly, we will continue to validate the technology in future studies.

In summary, we generated an algorithm to predict suicidal behavior and risk in individuals using Twitter. Our approach uses well-established theories and models of suicide to build effective neural networks that predict suicide ideation before suicidal thoughts are articulated. The model built is also effective in predicting suicide ideation up to three weeks preceding an event. Future studies can refine this algorithm by examining data from varied population groups and including measures from psychometrically validated scales on depression and suicide. An online-data-based predictive tool such as this holds hope not only for timely and personalized interventions but also has potential in tracking effectiveness of interventions at a population level. Future studies may evaluate the efficacy of regional scoring to facilitate targeting, assessment, and optimization of suicide prevention and postvention efforts in selected communities and should be studied further.

## Methods

### Collection of tweet database for SI cases and controls

Using the Tweepy package in python, we accessed the Twitter API, which allows access to the past 9 days of publicly available Twitter content for a given query. Beginning in September of 2016 and proceeding until June 2019, we performed weekly queries for the term “I suicide thinking OR planning” to find users expressing suicidal ideation. User timelines of individuals within these sets were subsequently downloaded using the Twitter API. At algorithm onset, individual user IDs were replaced by random unique identifiers and the names of identifying URLs and mention of other Twitter users was scrubbed from tweet content. At various times throughout the year, control tweets were gathered in a similar manner using the search term “I”. Notably, a fair portion of users identified by the “I suicide thinking OR planning” query were suicide prevention tweets or discussion of suicide related topics such as the movie “Suicide Squad”, and famous celebrity suicides, among others. Importantly, user timelines from these individuals or organizations were kept in the model (see below) as controls to improve the specificity towards actual SI tweets. No geographic parameters were selected for the identification of individuals, but in accordance with local copyright laws, tweets, and data from individuals listing a location anywhere inside the United Kingdom were blocked from being evaluated and deleted by the algorithm.

According to Canada’s Tri-Council Policy Statement: Ethical Conduct for Research Involving Humans—TCPS 2 Article 2.5, research on data “In the public domain and the individuals to whom the information refers have no reasonable expectation of privacy (non-intrusive, does not involve direct interaction between the researcher and individuals through the Internet)” is exempt from IRB/research ethics board (REB) review. This policy is consistent with US regulations where according to US Department of Health and Human Services Policy 45 CFR 46.104 under the heading ‘Exempt research, Secondary research for which consent is not required”, secondary research uses of identifiable private information or identifiable biospecimens are exempt if the identifiable private information or identifiable biospecimens are publicly available. Exemption for the need for REB review was confirmed through the Office of Research Ethics at the Royal’s Institute of Mental Health Research.

### Identification of SI tweets

As we collected a data set of tens of millions of tweets, we implemented a scanning strategy to first identify potentially suicidal tweets that would later be evaluated by a psychiatrist rater. Each user’s timeline was initially scanned for the presence of selected combinations of words appearing in a particular order. For a tweet to be selected for potential classification as an SI event, it would have to match at least one of the word patterns in Supplementary Table 1. All available tweets from individuals flagged as containing at least one SI event were subsequently scanned for the presence of the word “suicide” by the algorithm. Notably, in light of the popular use of the phrase, “I want to die” as referring to states of embarrassment, this phrase was not used as SI. All flagged tweets were subsequently evaluated by a psychiatrist to confirm if they appeared to represent SI at least 3 months following data collection and deemed “events”. When it was possible to assign a day to a past admission of suicide attempt by the rater, that day was included as an “event”, as it represents the suicide risk condition we sought to classify. In control individuals, a randomly selected tweet from the period of the user’s timeline was classified as an “event” so that later analyses comparing tweets preceding SI events in cases relative to controls could be performed. Most controls were not flagged by the scanning algorithm as having mentions of suicide; however, if a tweet was flagged for clinical consideration but deemed non-SI, it was kept as a separate event in the analysis stage of our study. Examples of these events include discussing suicide prevention or celebrity suicides.

### Identification of Individuals with admitted past history of suicide attempt (SA) or suicide plan (SAP)

In a similar manner to the above, all tweets were scanned with an algorithm flagging those tweets with words appearing in a specified order. Word selections and order appear in Supplementary Table 1. All flagged tweets were subsequently evaluated by a psychiatrist to confirm if the individual appeared to be a person with admitted past history of SA or suicide plan (SAP). Only those individuals with active Twitter accounts in January 2020 were included in the SAP analysis.

### Identification of individuals having died of suicide

We downloaded public Twitter profiles of *N* = 9 celebrities for whom death by suicide had been reported as the cause of death in the media. The date of death reported online was used as the “event” date for subsequent analysis.

### Sample demographics

In total, we collected a data set of 7,223,922 tweets. From these 6,385,079 were from individuals deemed controls and 838,843 derived from suicidal ideators. The frequency of tweets per day between cases and controls in the 365 days preceding an event were not different (Student’s t test; mean tweets per day cases: 2.61 ± 9.88, mean tweets per day controls: 3.34 ± 16.08, *p* = 0.29). Using the M3inference package in python^47^, we estimated the age and sex of each individual where possible. The algorithm combines a convolutional neural network assessment of user profile picture, user written description, and user name to estimate the probability of the user belonging to an age class of ≤18, between 20–29, 30–39, and over 40. For our analysis, we generated an individual level age estimate using the following Equation 1:

$${\mathrm{Age}} = \left( {{\mathrm{a}}18_{\mathrm{p}} \times 18} \right) + \left( {{\mathrm{a}}20{\mathrm{s}}_{\mathrm{p}} \times 25} \right) + \left( {{\mathrm{a}}30{\mathrm{s}}_{\mathrm{p}} \times 35} \right) + \left( {{\mathrm{a}}40_{\mathrm{p}} \times 45} \right),$$

where (a18_p_, a20s_p_, a30s_p_, and a40_p_) is the estimated probability per respective age group. For the sex estimate, individuals with a sex estimate probability ≥80% were binary coded as male or female, respectively. Of the 5920 individuals in the study, demographic estimates were obtainable for 4571.

### Random geographic sampling of county-wide Twitter data

Using the Twitter API, we downloaded 50 tweets on an hourly basis using the query term “I” paired with the central longitude and latitude and square root of the square mileage for each of ninety-two US counties for which the most recent 2017 county-wide suicide death rate data was available from the CDC wonder database (https://wonder.cdc.gov/). As such, tweets sampled should roughly derive from a circular area with a diameter equivalent to the width of each county. Tweets were collected during an initial period in August 2019 and during a replication period in October 2019. Using the CDC wonder database output, we analyzed data relative to age adjusted death rates listed for ICD-10 code GR113-124, “Intentional self-harm (suicide)”. For age-based analyses, non-age adjusted crude death rates for GR113-124 were taken as representative of suicide death rates per age group. Given the scope of randomly sampled individuals in this analysis, we did not attempt to use the M3inference package to infer age and sex of our generated data set.

### Algorithm generation

The algorithm consisted of a tiered process to achieve two objectives. The first objective was to generate a method to classify SI cases from controls and the second objective was to generate a method to assess when flagged individuals are likely to be at risk. For objective one, the algorithm structure involves first converting text based tweet content into scores representative of psychological constructs using neural networks and subsequently training a random forest on the neural network derived data in a training set of cases and controls to predict case status. For objective two, tweet level random forest prediction scores generated over a user’s timeline are evaluated for alterations in the frequency of high scores relative to the user’s average pattern.

### Generation of neural networks for psychological scoring of sentence content

Neural networks were generated to reduce text content into a score ranging between 0 and 1 for psychological constructs of burden, loneliness, stress, anxiety, insomnia, and depression. To generate neural networks, query terms according to Supplementary Table 2 were input into Twitter and no more than 1000 tweets were collected from the Twitter API. Three separate models for depression were generated (depression 1, depression 2, and depression 3). While we hoped to use a relatively agnostic approach to generation of neural network training data from the Twitter Sphere, for the case condition we sought to refine those included tweets to those depicting a relatively negative as opposed to positive sentiment. In this way, we hoped to avoid confounding statements such as, “I am hopelessly in love with him” being incorporated into the “hopelessness” model, for example. To achieve this, sentiment analysis using the textBlob package in python was used to filter tweets such that psychological term tweets had a subjectivity score ≥0.5 and a polarity score <0. Comparator (control) tweets were filtered on a sentiment subjectivity score ≥0.5 and polarity >0. Notably, the above average subjectivity score filter was used to bias training data to those data representing subjective as opposed to objective view points. These terms were then binary coded using a bag of words approach and used to train neural networks with a single hidden layer consisting of 20 neurons, with back propagation (100,000 epochs, batch size of 1) using logistic stochastic gradient decent and alpha of 0.001. Neural network hyper parameter optimization utilized k-fold cross validation across a range of neuron hidden layer sizes using the MPLclassifier function in the sklearn package (version 0.21.3) with neuron sizes of *N* = 10, 20, 30, 40. Across all models, mean k-fold AUC values were equivalent across hidden neuron sizes between 20, 30, and 40 neurons (IE: insomnia model: mean AUC = 0.97) but decreased at 10 neurons (IE: insomnia model: mean AUC = 0.91). Subsequent models were trained with hidden layer sizes of 20 neurons.

Neural network code was written in python 3.6.8. Using the same training sets, we also trained linear kernel support vector machine (SVM) models for each psychological construct using the sklearn (version 0.21.3) package in python.

### Validation of neural networks

The neural networks constructed were designed to read text and infer psychological weights across a range of constructs including stress, loneliness, burdensomeness, hopelessness, depression, anxiety, and insomnia. While neural networks were trained from the tweets of people referencing query words related to these concepts such as “burden”, we sought to validate their ability to reflect these constructs. To that end, we adapted psychometrically validated psychological scales by binary coding question content in relevant scales where the original responses were amenable to such adaptation, such as in true vs. false response (Supplementary Table 3). For example, in the perceived stress scale^48^, the sentence, “I find that I cannot cope with all the things that I have to do”, would be coded as a 1 while the sentence, “I am able to control irritations in my life” would be coded as a 0. In this way, we could use neural networks to score each scale and generate the area under the receiver operating characteristic curve (AUC) to derive their accuracy at capturing these metrics.

### SI model cross validation strategy

We employed two strategies for model training and independent validation. The primary validation strategy involved allocating approximately 50% of the entire sample into a training and test sample data set such that roughly equal numbers of cases and controls would be selected from each month, where the most recent “event” day occurred within that month (training set: *N* cases = 283, *N* tweets = 512,526, *N* controls = 2655, *N* tweets = 3,518,494, test set: *N* cases = 277, *N* tweets = 326,317, *N* controls = 2691, *N* tweets = 2,866,585). The rationale for matching within months involved controlling for variation of seasonal effects, changes in lexicon, and events like hurricanes, stock market fluctuations, and elections. The proportion of SI in each set was similar to the observed rate of 9.2% observed in a cross-national study of SI rates in the general population^33^. Age and sex distributions were not different between the training and test sets (Student’s *t*-test: mean training age = 29 ± 7.7, mean testing age = 29 ± 7.8, *p* = 0.22; Student’s *t*-test: mean training male probability = 0.56 ± 0.45, mean testing male probability = 0.57 ± 0.45, *p* = 0.5). As a second validation strategy, we split the sample by year, building the training and test sets from individuals where the most recent “event” occurred prior to and after August 2018, respectively (training set: *N* cases = 265, *N* tweets = 532,811, *N* controls = 2422, *N* tweets = 2,787,152; test set: *N* cases = 278, *N* tweets = 329,162, *N* controls = 2911, *N* tweets = 3,374,966).

### Generation of random forest classifiers

Using the training sets above, we trained a series of random forest classifiers across two scenarios. Training set data input into the random forests consisted of (1) inputting *N* = 9 neural network derived model scores in addition to the subjectivity and polarity metrics from sentiment analysis and (2) inputting *N* = 9 neural network derived model scores independent of sentiment analysis metrics to understand the added value of neural network scores. Across all scenarios, we employed a bootstrap aggregating approach to generate equivalent numbers of cases and controls per generated model for a total of *N* = 10 models, where all cases from the training sets remained in the model but randomly selected equivalent numbers of control cases were utilized, where the 10th group was comprised partially of controls used previously to even the groups. Averaging was used to combine the model outputs, generating a quantitative score between 0 and 1 for each tweet assessed in the test sets. For each training set of bootstrap aggregated controls and cases, we optimized model hyper parameters using a grid search technique using the GridSearchCV function in sklearn package (version 0.21.3) with the default *N* = 10 estimators, range of maximum depth of 2, 4, 6, 8, 10, 100, and 500, and employing k-fold cross validation across five k-folds, identifying no max depth as optimal. To validate that the models were not driven by outliers, we employed cross validation across five K-folds on a random forest generated on the training set and generated a mean accuracy of 0.83 ± 0.040. K-fold cross validation with *N* = 100 estimators as is the default in later versions of sklearn returned virtually identical cross validation scores using the cross_val_score function. All subsequent bootstrap aggregated random forests were trained with *N* = 10 estimators and random state seed of 0.

### Model assessment

All random forest model scenarios were assessed for their ability to distinguish cases from controls by averaging tweet level random forest generated scores in test set individuals in two scenarios. In scenario 1, we assessed mean model score during a period at least 1 day prior to the “event” day (day of voicing of an SI event in cases; randomly selected day or non SI mention of suicide topic in controls) and at least seven days after any previous event to generate an overall predictive accuracy score. This scenario was also compared to the prediction of SI cases and controls using only sentiment polarity scores as a benchmark comparison. In scenario two, a sliding window approach was employed to combine and average tweet level random forest model scores prior to the event day across a range of window ranges including 4, 7, 14, and 21 days.

### Temporal analysis

Temporal analysis was performed by limiting the data set to SI events for which at least 120 days (4 months, as a larger proportion of individuals had this amount of data) worth of data existing prior to the event and establishing a threshold unique to each individual comprised of the mean model score for the period. The frequency of model scores above this threshold (denoted frequency score) within a sliding window was quantified for each day. We evaluated a range of window spans including 4, 7, 14, and 21 days. The the total number of tweets per window was tabulated and devided it by the window span (denoted baseline) to acount for variation in individual specific tweeting frequency. We then evaluated whether or not the frequency score exceeded the baseline temporally closer to the actual SI event (denoted peak). Over the entire dataset, we then statiscally assessed if peaks occur within a range of times from 5 to 120 days using logistic regression.

### Statistical analysis

All statistical tests were performed in R (http://www.r-project.org/). Using an Anderson–Darling test from the nortest package, all distributions of data that rejected the null hypothesis of normality were subsequently evaluated with non-parametric tests. All statistical tests performed were two-tailed and a *p* < 0.05 was considered significant. No correction for multiple comparisons were required. Unless otherwise specified ± denotes the standard deviation. AUC metrics were generated using the pROC package in R. For AUC tests, significance was determined using a Monte Carlo card sorting approach to assess the null distribution of AUC values upon randomizing diagnostic status across *N* = 10,000 permutations.

### Reporting summary

Further information on experimental design is available in the Nature Research Reporting Summary linked to this article.

## Supplementary information

Supplementary Information

Reporting Summary

## Data Availability

In accordance with Twitter policies of data sharing, data used in the generation of the algorithm for this study will not be made publicly available.

## Code availability

Due to the sensitive and potentially stigmatizing nature of this tool, code used for algorithm generation or implementation on individual Twitter profiles will not be made publicly available. All python scripts were created in python version 3.6.8 and machine learning algorithms utilized the sci-kit (sklearn) version 0.21.3. Specific parameters used to generate, test, and process the current dataset are mentioned in detail in the “Methods” section.
