# Supplementary material for: A machine learning approach predicts future risk to suicidal ideation from social media data
Source: NPJ Digit Med. 2020 May 26;3:78. doi: 10.1038/s41746-020-0287-6 (PMC7250902; doi:10.1038/s41746-020-0287-6)
Supplement: Supplementary file 1 — Supplementary Information [file 41746_2020_287_MOESM1_ESM.pdf]

Supplementary Note 1

In the analysis of county-wide suicide death rate data, our data represented a hierarchically ordered system in which individual tweets in a day and over multiple days were nested within counties. To avoid the atomistic fallacy, we used a hierarchical linear model to examine if SI scores from individual tweets within a county reflected death by suicide rates for that county. We first regressed SI scores on county with inclusion of a random intercept to capture the variability in SI risk for each county. The conditional modes derived (equivalent to best linear unbiased predictors) give an estimate of the extent to which the death by suicide rate differs for a specific county compared to the average for all counties. Furthermore, as SI risk may vary with time and scores over a single day may not capture such changes adequately, we combined information gathered over twenty days in August and September 2019. We modelled the average effect of time (in days) on SI scores within each county by regressing SI scores on time and including a random intercept as well as random slope for each county. This allowed us to model between-county differences in average SI scores over multiple days as well as the change in SI scores over time. Next, we examined the associations between the conditional modes derived and age-adjusted death by suicide rates using generalized linear regressions (with a natural log link function). We used the negative binomial model due to over dispersion in our data. Results showed that SI scores from tweets over the twenty days in each county were associated with county-wide death rates (IRR = 1.27, SE = 1.10, $p = .015$). We next used subsamples of our data to understand the minimum number of days required to generate aggregated SI scores that would be predictive of death by suicide rates and determined minimum of sixteen days was required (IRR = 1.91, SE = 1.22, $p = .0013$)(Figure 6A). Further, the rate of change in SI scores during this time also predicted death rates (IRR = 1.94, SE = 1.22, $p = .0010$).

To validate our findings, we collected and scored additional Twitter data over twenty-nine days in September and October 2019 and observed significant associations between mean county SI scores and county-wide death rates (tau = 0.31, $p = 1.16 \times 10^{-5}$)(Figure 5B). Similarly, the hierarchical linear model analysis exhibited significant associations of SI scores over these twenty-nine days (IRR = 1.33, SE = 1.06, $p = 4.48 \times 10^{-6}$) as well as change in SI score during this time (IRR = 1.39, SE = 1.06, $p = 1.86 \times 10^{-7}$) with county-wide death rates. Restricting the analyses to the first sixteen days only showed associations between county-wide SI scores and death rates (IRR = 1.24, SE = 1.08, $p = .0038$) as well as between change in SI scores over the 16 days and death rates (IRR = 1.16, SE = 1.08, $p = .048$). Analyses restricted to the last sixteen days showed comparable results. Further sub-samplings showed that in this data from September/October 2019, SI scores over eight days were sufficient to predict county-wide death rates (multiple subsets of eight days; all $p < 0.05$).

Supplementary Note 2

We next examined the discrepancy in minimum number of days required to predict death by suicide rates between the original (August/September) and replication (September/October) datasets, that is, with data over twenty days and twenty-nine days, respectively; while SI scores over at least sixteen days were needed to consistently predict suicide death rates in the August/September 2019 data, only eight days were needed in the September/October 2019 data. We found that for the August data, scores from the later eight days, but not the initial eight days, could be associated with county-wide death rates, indicating that an eight day period in this month did not show consistent results. However, the early August time-period during which Twitter data was collected, followed the 2019 El Paso and 2019 Dayton shooting incidents, which may have led to widespread changes in tweeting patterns. In support of this hypothesis, mean SI score across all counties over three days in the week following these incidents (0.80), compared to mean SI score on other days in August (0.81), was significantly lower ($t = -2.10$, $p = .035$). Furthermore, between-county variability in SI scores (or standard errors of SI scores for each county) was higher in the time-period after the shooting than during other days in August (mean of post-shooting time = .004, mean of other days in August = .002, $t = 41.19$, $p = 2.2 \times 10^{-16}$), suggesting that model derived scores may be sensitive to acute but widely impactful events when analyzing short periods of time.

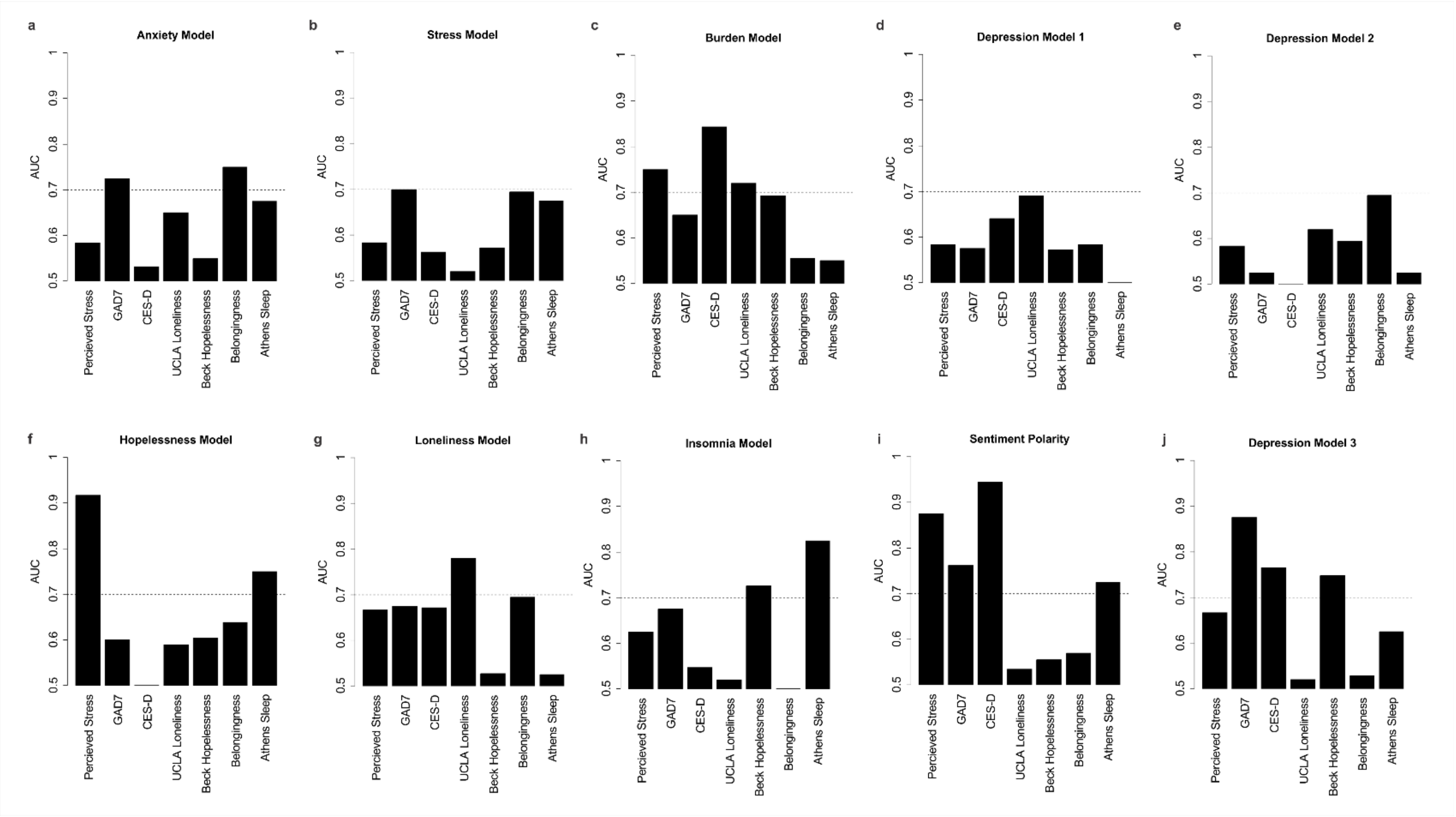

Supplementary Figure 1. SVM model performance to rate binary construct scales

Bar plots of the AUC of the ROC curve (y axis) for SVM based classification of binary statement data adapted from various scales (x axis) psychometrically validated to rate psychological constructs for the anxiety model (a), stress model (b), burden model (c), depression model 1 (d), depression model 2 (e), hopelessness model (f), loneliness model (g), insomnia model (h), sentiment analysis polarity metric (i), depression model 3 (j). A horizontal dashed line depicts an AUC of 70% accuracy. Binary adaptations of scales appear in Supplementary Table 1. The scale-based validation showed that networks trained on words associated with the query ‘depression’ had variable performance across a number of psychological constructs. Although, depression represents the largest global burden, it is likely that the batch of gathered Tweets collected using this query is inadequate to capture all references to depression on the Twitter sphere at a single training instance. As such, multiple samplings across multiple days is likely necessary to generate an adequate training sample to reflect the constructs inherent in depression. While we opted instead to generate multiple models for the query term ‘depression’, we noted that model performance was optimal when generating individual models as opposed to combining all potential depression training tweets into a single training set. As depression is a heterogeneous disorder with a range of possible symptoms leading to a diagnosis, it is possible that varied networks trained on a ‘depression’ query would reflect the heterogeneity inherent in this psychological construct. However, networks trained together may fail to generate a ‘true’ model due to its inability to capture such heterogeneity.

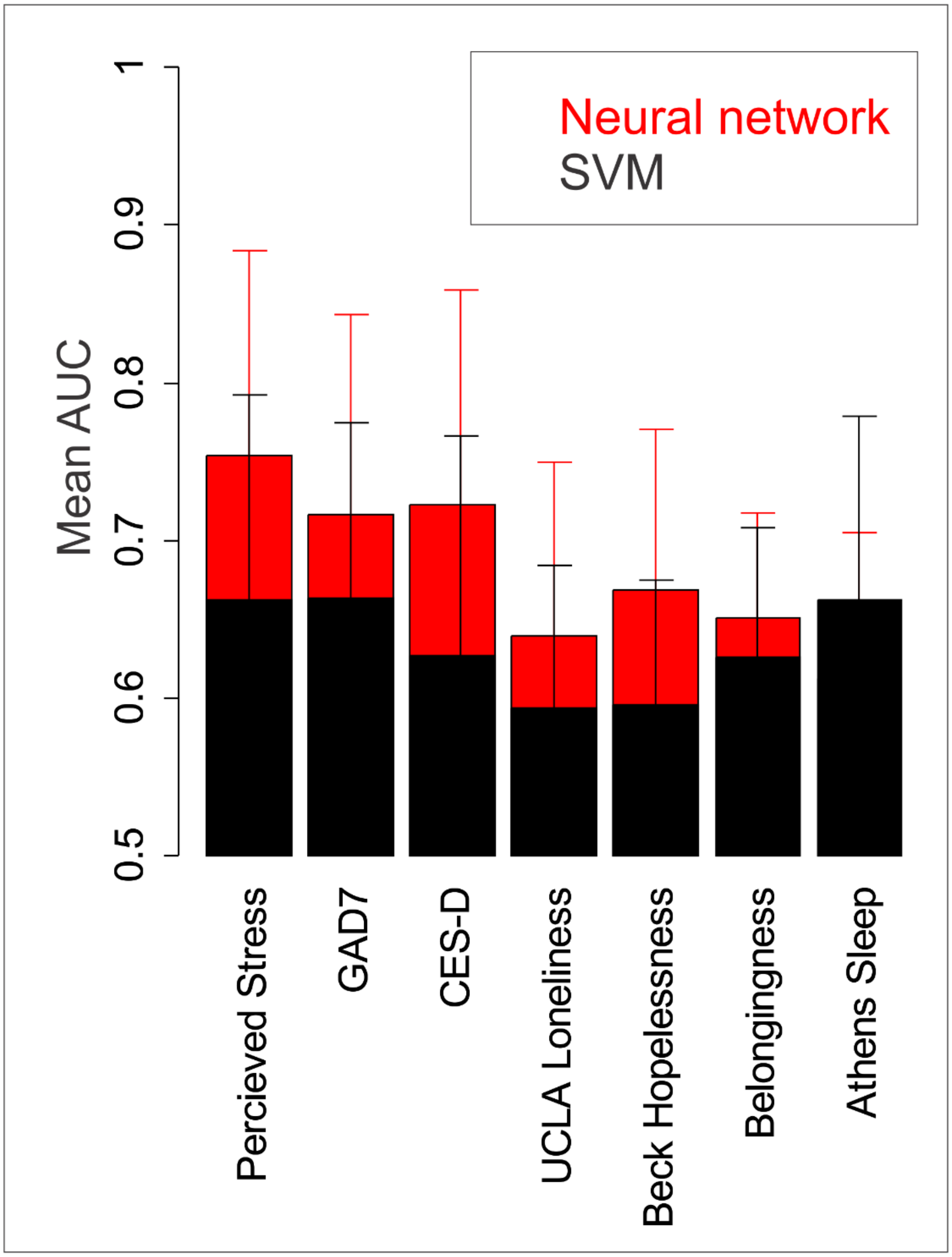

Supplementary Figure 2. Comparison of neural network vs. SVM performance to rate binary construct scales
A bar plot of the mean AUC (y axis) generated for classification of binary statement data adapted from scale data (x axis) for data derived from neural network classifiers (red) and SVM classifiers (black). Vertical Ts represent the standard deviation.

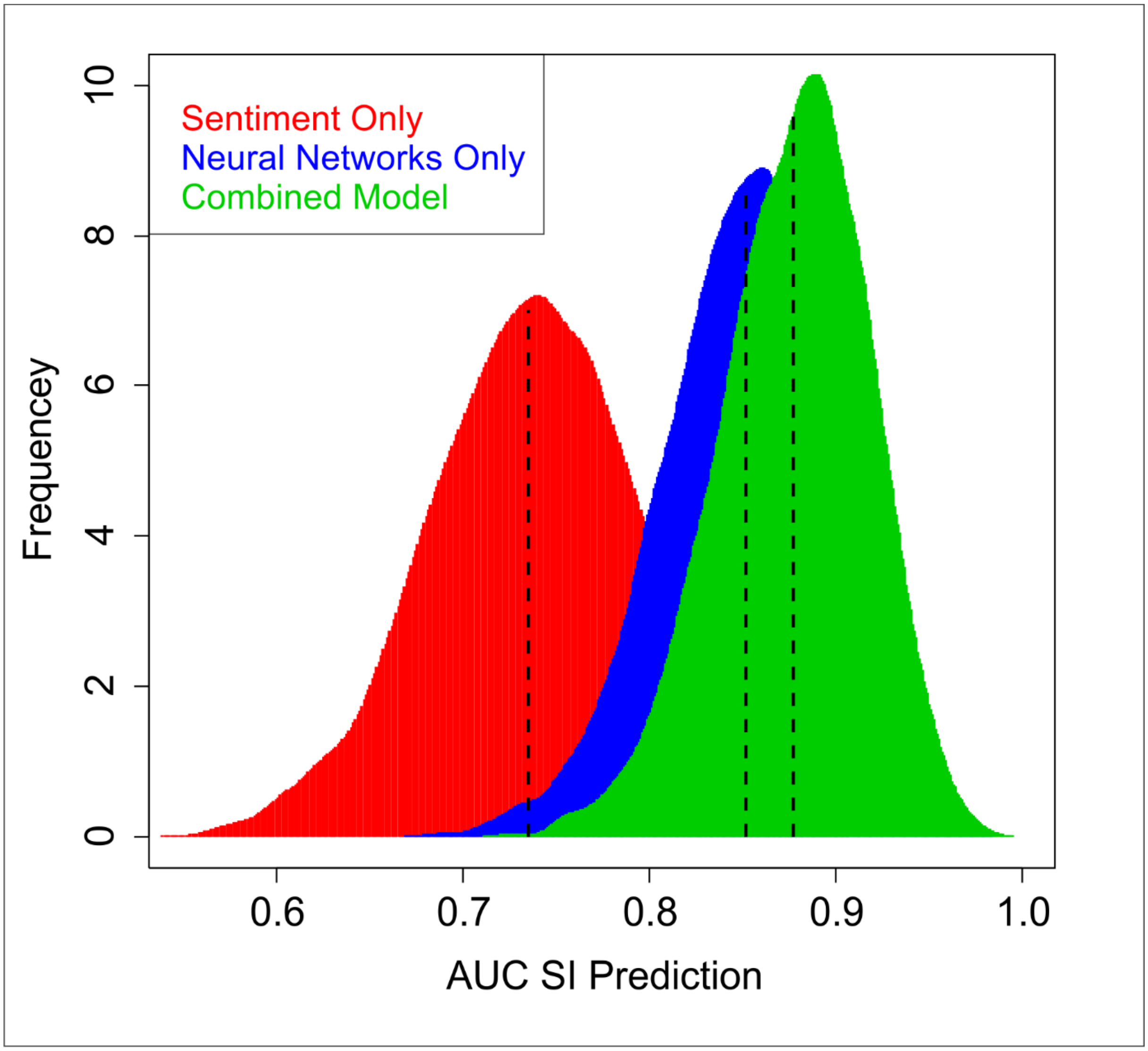

Supplementary Figure 3. Permuted Subsampling of SI Prediction Models

A density plot depicting the distribution of AUC values obtained from predicting N=10,000 permutations of random sub samplings of 50 SI cases from 50 controls. The red distribution represents AUC values obtained using sentiment analysis based polarity scores alone (mean AUC= 0.74 ± 0.055). The blue distribution represents AUC values obtained using the bootstrap aggregated random forest strategy on the 8 psychological construct neural networks alone (mean AUC= 0.85 ± 0.045). The green distribution represents AUC values obtained using the bootstrap aggregated random forest strategy on the 8 psychological construct neural networks and sentiment polarity score together (mean AUC= 0.88 ± 0.04). Vertical black dashed lines represent the mean of each distribution.

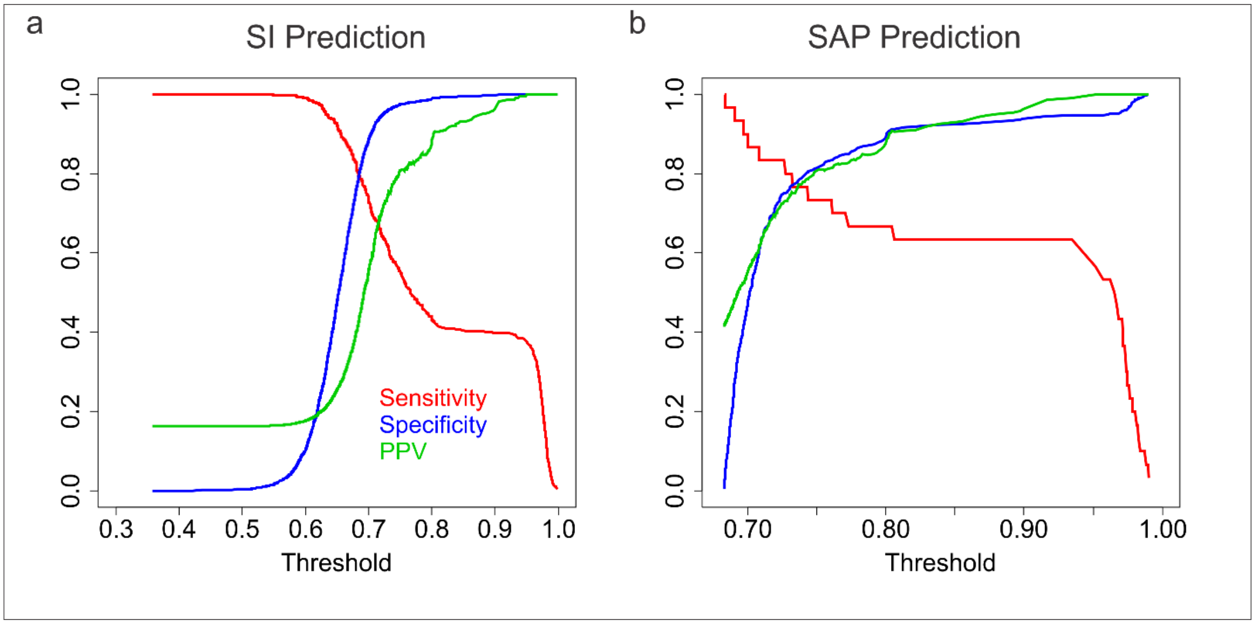

Supplementary Figure 4. Model performance by score threshold

a.)A plot of the sensitivity (red), 1-specificity (blue), and positive predictive value (green) (y axis) as a function of threshold scores (x axis) to predict SI events from control events. A threshold score of 0.683 achieves maximal sensitivity and specificity and returns a posterior probability of 40% increased risk. b.) A plot of the sensitivity (red), 1-specificity (blue), and positive predictive value (green) (y axis) as a function of threshold scores (x axis) to predict SI events in N=30 SAP individuals from N=426 non-SAP individuals in people with a threshold score ≥ 0.683. A threshold score of 0.731 achieves maximal sensitivity and specificity and returns a posterior probability of 75.2% increased risk of being a SAP individual with a score above this threshold.

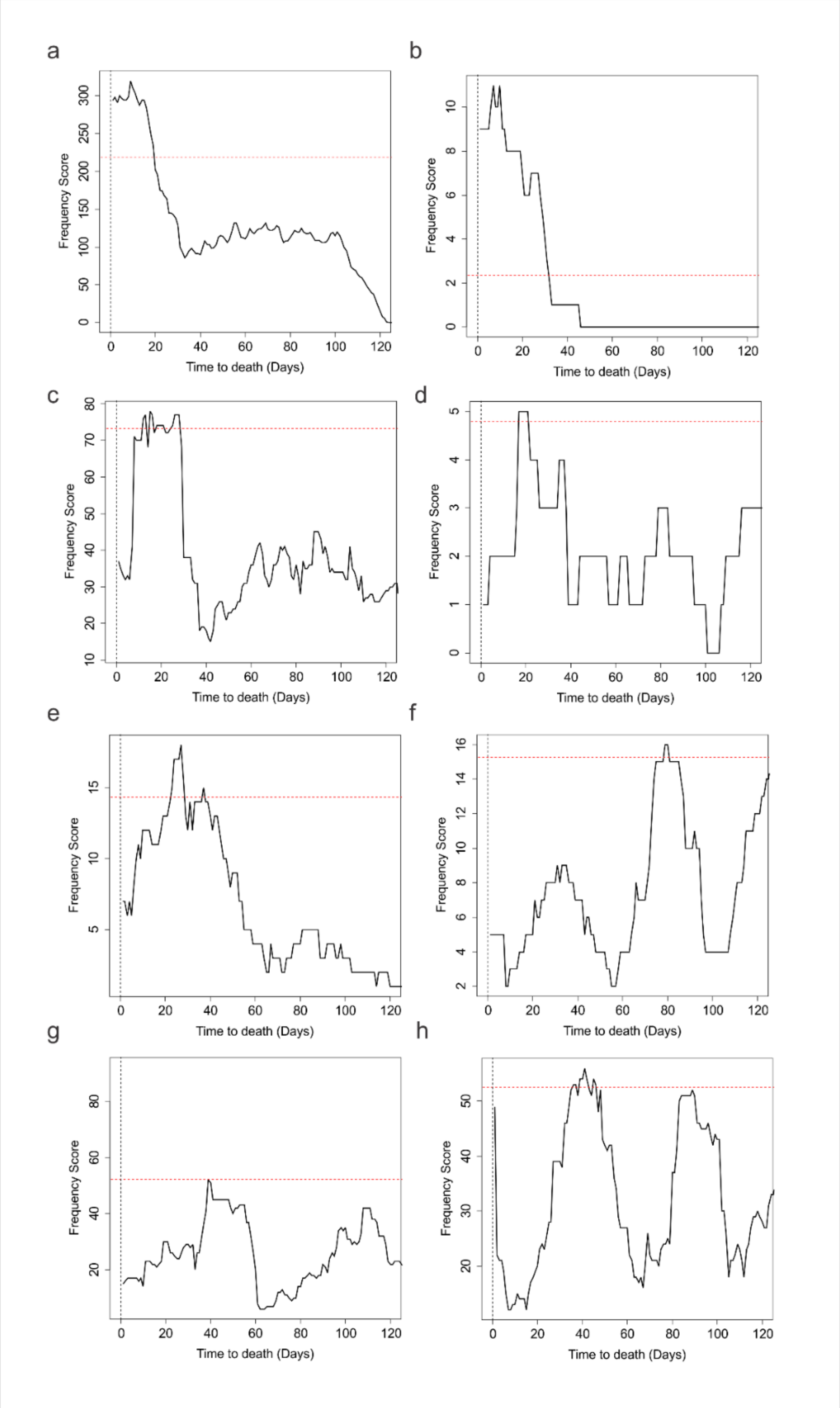

Supplementary Figure 5. Individual frequency score thresholds and profiles for suicide decedents
Plots depicting the frequency score (y axis) as a function of time from death by suicide for N=8 suicide decedents (a,b,c,d,e,f,g,h) derived from data generated using a 21 day span.

Supplementary Table 1: *Ordered word screening for human evaluation*

SI tweet criteria

I	am	planning	to	die	
I	am	going	commit	suicide	
I	attempted	suicide			
my	suicide	attempt			
I	planning	to	end	my	life
I	planned	to	end	my	life
I	planned	die			
I	planning	to	kill	myself	
I	tried	to	kill	myself	
I	thinking	suicide			
I	can't	go	on	living	
I	want	to	die		
I	want	to	kill	myself	
I	going	to	kill	myself	
I	have	plan	kill	myself	
I	contemplating	suicide			
I	planning	suicide			
I	feeling	suicidal			
I	having	suicidal	thoughts		
I	am	planning	to	die	
I	am	going	commit	suicide	
I	want	to	commit	suicide	
I	want	to	end	my	life
I	planning	to	end	my	life
I	want	to	end	my	life
I	planning	to	kill	myself	
I	thinking	how	to	kill	myself
I	thinking	suicide			
I	can't	go	on	living	
I	want	to	die		
I	want	to	kill	myself	
I	going	to	kill	myself	
I	contemplating	suicide			
I	planning	suicide			
I	feeling	suicidal			
I	having	suicidal	thoughts		

Past SA or suicide plan tweet criteria

I	am	planning	to	die	
I	attempted	suicide			
my	suicide	attempt			
I	planning	to	end	my	life
I	planned	to	end	my	life
I	planned	die			
I	planning	to	kill	myself	
I	tried	to	kill	myself	

I	have	plan	kill	myself
I	planning	suicide		

Supplementary Table 2: *Twitter Query Terms for Neural Network Training Set Generation*

Model	Query Date	Case Query	Query Date	Control Query
Burden Model	9/27/2017	I am a burden	9/27/2017	I am happy OR I am good
Loneliness Model	9/27/2017	I am lonely	9/27/2017	I am happy OR I am good
Stress Model	10/5/2017	I am stressed OR stress	9/27/2017	I am happy OR I am good
Depression Model 1	5/14/2018	I am depressed	9/27/2017	I am happy OR I am good
Anxiety Model	6/19/2018	I am anxious OR I am nervous	9/27/2017	I am happy OR I am good
Depression Model 2	6/26/2018	I am depressed	9/27/2017	I am happy OR I am good
Hopelessness Model	6/28/2018	I hopeless	9/27/2017	I am happy OR I am good
Depression Model 3	7/10/2018	I am depressed	9/27/2017	I am happy OR I am good
Insomnia Model	7/22/2019	I am not sleeping OR I can't sleep OR insomnia	7/22/2019	good sleep

Supplementary Table 3: *Binary Psychological Construct Scale Adaptations*

**Binary Perceived Stress Scale**	
1	I get upset because of something that happened unexpectedly
1	I feel I am unable to control the important things in my life
1	I often feel nervous and "stressed"
1	I find that I can not cope with all the things that I have to do
1	I have been angered because of things that were outside of my control
1	I feel difficulties are piling up so high that I could not overcome them
0	I feel confident about my ability to handle my personal problems
0	I feel things are going my way
0	I am able to control irritations in my life
0	I feel that I am on top of things
**Binary Athens Sleep Scale**	
1	It takes a long time to fall asleep after turning off the lights
1	I do not sleep at all after turning off the lights
1	I wake up much earlier than I want
1	I have very insufficient sleep or do not sleep at all
1	I have very poor quality of sleep
1	I have a satisfactory quality of sleep
1	I have decreased functioning during the day
1	I am intensely sleepy during the day
0	I do not wake up during the night
0	I get enough sleep
0	I have a normal sense of well being during the day
0	I function normally during the day
0	I am not sleepy during the day
**Binary General Belongingness Scale**	
1	I feel like an outsider
1	I feel as if people do not care about me
1	Because I do not belong, I feel distant during the holiday season
1	I feel isolated from the rest of the world
1	When I am with other people, I feel like a stranger
1	Friends and family do not involve me in their plans
0	When I am with other people, I feel included
0	I have close bonds with family and friends
0	I feel accepted by others
0	I have a sense of belonging
0	I have a place at the table with others
0	I feel connected with others
**Binary CES-D**	
1	I was bothered by things that usually don't bother me
1	I did not feel like eating; my appetite was poor
1	I felt that I could not shake off the blues even with help from my family or friends
1	I had trouble keeping my mind on what I was doing
1	I felt depressed

1	I felt that everything I did was an effort
1	I thought my life had been a failure
1	I felt fearful
1	my sleep was restless
1	I talked less than usual
1	I felt lonely
1	People were unfriendly
1	I had crying spells
1	I felt sad
1	I felt people dislike me
1	I could not get going
0	I felt that I was just as good as other people
0	I felt hopeful about the future
0	I was happy
0	I enjoyed life
**Binary GAD7**	
1	I am feeling nervous
1	I am feeling anxious
1	I am on edge
1	I can't stop worrying
1	I can't relax
1	I worry too much about different things
1	I become easily annoyed
1	I become irritable
1	I feel afraid something awful might happen
1	I am restless and it is hard to sit still
0	I can control my worrying
0	I never have trouble relaxing
0	I take it easy
0	I find it easy to relax
**Binary UCLA Loneliness Scale**	
1	I lack companionship
1	There is no one I can turn to
1	I am no longer close to anyone
1	My interests and ideas are not shared by those around me
1	I feel left out
1	My social relationships are superficial
1	No one really knows me well
1	I feel isolated from others
1	I am unhappy being so withdrawn
1	People are around me but not with me
0	I feel in tune with the people around me
0	I do not feel alone
0	I feel part of a group of friends
0	I have a lot in common with the people around me
0	I am an outgoing person
0	There arc people I feel close to

0	I can find companionship when I want it
0	There are people who really understand me
0	There are people I can talk to
0	There are people I can turn to
**Binary Beck Hopelessness Scale**	
1	I just don't get the breaks, and there's no reason to believe I will in the future
1	I never get what I want so it's foolish to want anything
1	There's no use in really trying to get something I want because I probably won't get it
1	I might as well give up because there's nothing I can do about making things better for myself
1	It is very unlikely that I will get any real satisfaction in the future
1	All I can see ahead of me is unpleasantness rather than pleasantness
1	my past experiences have prepared me well for my future
0	I look forward to the future with hope and enthusiasm
0	I have great faith in the future
0	When things are going badly, I am helped by knowing that they can't stay that way forever
0	I can look forward to more good times than bad times
0	In the future, I expect to succeed in what concerns me most
0	When I look ahead to the future, I expect I will be happier than I am now
0	I have enough time to accomplish the things I most want to do
0	I happen to be particularly lucky and I expect to get more of the good things in life than the average person
0	I can't imagine what my life would be like in 10 years
0	I don't expect to get what I really want
0	Things just won't work out the way I want them to
0	The future seems vague and uncertain to me
0	my future seems dark to me
